# Vulnerability of *Tritia reticulata* (L.) early life stages to ocean acidification and warming

**DOI:** 10.1038/s41598-020-62169-7

**Published:** 2020-03-24

**Authors:** Isabel B. Oliveira, Daniela B. Freitas, Joana G. Fonseca, Filipe Laranjeiro, Rui J. M. Rocha, Mariana Hinzmann, Jorge Machado, Carlos M. Barroso, Susana Galante-Oliveira

**Affiliations:** 1 CESAM, Department of Biology, University of Aveiro, Campus Universitário de Santiago, 3810-193 Aveiro, Portugal; 2 CIIMAR - Interdisciplinary Centre of Marine and Environmental Research, Terminal de Cruzeiros de Leixões, Av. General Norton de Matos s/n, 4450-208 Matosinhos, Portugal; 3 Laboratory of Applied Physiology, ICBAS, University of Porto, Rua de Jorge Viterbo Ferreira 228, 4050-313 Porto, Portugal

**Keywords:** Climate-change impacts, Scanning electron microscopy, Non-model organisms

## Abstract

Ocean acidification and warming (OA-W) result mainly from the absorption of carbon dioxide and heat by the oceans, altering its physical and chemical properties and affecting carbonate secretion by marine calcifiers such as gastropods. These processes are ongoing, and the projections of their aggravation are not encouraging. This work assesses the concomitant effect of the predicted pH decrease and temperature rise on early life stages of the neogastropod *Tritia reticulata* (L.), a common scavenger of high ecological importance on coastal ecosystems of the NE Atlantic. Veligers were exposed for 14 days to 12 OA-W experimental scenarios generated by a factorial design of three pH levels (targeting 8.1, 7.8 and 7.5) at four temperatures (16, 18, 20 and 22 °C). Results reveal effects of both pH and temperature (T °C) on larval development, growth, shell integrity and survival, individually or interactively at different exposure times. All endpoints were initially driven by pH, with impaired development and high mortalities being recorded in the first week, constrained by the most acidic scenarios (pH_target_ 7.5). Development was also significantly driven by T °C, and its acceleration with warming was observed for the remaining exposure time. Still, by the end of this 2-weeks trial, larval performance and survival were highly affected by the interaction between pH and T °C: growth under warming was evident but only for T °C ≤ 20 °C and carbonate saturation (pH_target_ ≥ 7.8). In fact, carbonate undersaturation rendered critical larval mortality (100%) at 22 °C, and the occurrence of extremely vulnerable, unshelled specimens in all other tested temperatures. As recruitment cohorts are the foundation for future populations, our results point towards the extreme vulnerability of this species in case tested scenarios become effective that, according to the IPCC, are projected for the northern hemisphere, where this species is ubiquitous, by the end of the century. Increased veliger mortality associated with reduced growth rates, shell dissolution and loss under OA-W projected scenarios will reduce larval performance, jeopardizing *T. reticulata* subsistence.

## Introduction

The netted whelk, *Tritia reticulata* (Linnaeus, 1758) is a common neogastropod, ubiquitous in the North East Atlantic shore –along the European coastline, from the Canaries to Norway– and throughout the Mediterranean, Baltic and the Black Seas^1^. Its occurrence extends from the low water of spring tides to sublittoral depths down to at least 40 m, along the open coast and the outer parts of estuaries. Adults spawn from mid-winter to summer, laying egg capsules onto hard substrates^2,3^. Inside the capsule, organisms pass through a trochophore larval stage before hatching as veliger, roughly after one month of development^1,3^. The veliger stage corresponds to the free-swimming, planktonic larval period, and is anatomically characterized by the presence of a shell, foot and velum. Depending mainly on the temperature, this stage may last between 1 to 3 months as larvae reaches competency before settling to the substrate, and metamorphosing to became benthic^1,4^. Due to its scavenger nature, netted whelks play an important role on the environment, leading to nutrients and energy transfer across different ecosystems, as well as on the carbon cycling, distribution and sequestration into the oceans^5^.

Oceans’ sustainability, on which life on the planet also depends, is now a top priority of the international agenda as a number of threats known to cause irreparable damage to both the Earth’s climate and to marine ecosystems have been identified. Among them are ocean acidification (OA) and warming (W), phenomena recognized as being aggravated by human activities, particularly those involving greenhouse gas (GHG) emissions such as carbon dioxide (CO_2_). The rise of the atmospheric carbon dioxide from anthropogenic emissions is known to be a critical problem directly linked to an increase on the ocean temperature and acidity^6^. The latest long-term projections of OA-W, to occur by the end of the 21^st^ century, were presented in the Fifth Assessment Report (AR5) of the Intergovernmental Panel on Climate Change (IPCC) considering several Representative Concentration Pathway (RCP) scenarios^7^: under RCP8.5 – scenario without additional efforts to constrain current emissions, close to the so called business-as-usual path^7,8^ – the sea surface temperature (SST) might globally rise at a rate >0.1 °C per decade, accounting for increases from about 1 to above 3 °C^9,10^, while sea surface pH, that decreased since the Industrial Revolution at a rate of up to 0.0024 units yr^−1^, might fall off additional 0.3 to 0.5 units in the northern hemisphere^11^. Nevertheless, and despite the meritorious attempt to predict future OA-W, it is unambiguous that conditions greater than the actual SST and pH global averages have been experienced in many regions and seasons^11–13^ and that the projected trends are likely to continue, or even accelerate, in the upcoming decades, in the absence of effective mitigation measures to constrain GHG emissions and human-induced climate change^13^.

OA-W has a significant impact on marine invertebrates and the ecosystems they inhabit^14–16^. Beyond the physical properties, these phenomena alter seawater carbonate chemistry affecting both carbonate secretion by marine calcifiers such as gastropod molluscs^17–19^, and its dissolution, with increased vulnerability of younger shells usually composed of more soluble calcium carbonate (CaCO_3_) polymorphs^20,21^. Thus, the panorama for early life stages is of particular concern as their sensitivity to OA-W may be the bottleneck for the species subsistence^22–25^.

Most studies performed on calcifying organisms’ early ontogeny concerns solely the effect of the pH decrease^25–31^. In fact, OA is thought to be a determinant factor on the formation of biogenic CaCO_3_ as it decreases carbonate ions concentration. Albeit a certain controversy on how the saturation state of CaCO_3_ polyforms may influence calcification^32,33^, it is generally agreed that a higher energy supply is needed in order to manipulate the physical chemistry at the calcification site. Besides, OA has been linked to increased hypercapnia (increased CO_2_/acidosis), a condition that may compromise the energy homeostasis and metabolism^34,35^. Temperature also plays a very important role on the aerobic metabolism of poikilothermic organisms, known to be temperature-dependent: it increases with the rising temperature to cope with the high-energy demand to maintain cellular functions, until it can no longer do so^36^. When this state is reached, the imbalance on the energy budget may eventually lead to decreased development, growth, and ultimately survival^36,37^. The assessment of the combined effects of OA-W is, therefore, essential, as marine organisms are being subject to these phenomena simultaneously rather than independently. In fact, significant combined effects of OA-W in marine calcifiers’ early life stages are emerging on the scientific literature (e.g.^19,24,38–41^). Most of the studies describe additive negative effects of both stressors, whereas antagonistic and, less commonly, synergistic effects have also been reported^42,43^.

Here we investigate the concomitant effect of acidification and warming processes on *T. reticulata* larval development, growth, shell integrity and survival at 12 OA-W experimental scenarios, established as series of progressive environmental conditions based on IPCC near to long-term projections for the oceans in the northern hemisphere, conditions to which this species is likely to be exposed in its natural habitat over time, in an attempt to foresee its performance and resilience to future OA-W.

## Materials and Methods

### Experimental setup and design

The Experimental Life Support System (ELSS) available at the Centre for Environmental and Marine Studies of the University of Aveiro (Portugal) was used to expose early life stages of the netted whelk, *T. reticulata* to 12 OA-W experimental scenarios for 14 days. The ELSS was developed by Coelho *et al*.^44^ as a modular construction, allowing different configurations to address specific questions using microcosm simulation of climate change, namely warming, and CO_2_ induced acidification in coastal and estuarine environments. Briefly, it consists of four autonomous saltwater reservoirs (see Fig. 1 in Coelho *et al*.^44^) in which pH is independently manipulated through CO_2_ bubbling controlled by feedback pH control systems (V2 control pH controller/monitor and V2 pressure regulator pro, TMC, UK), from which water is pumped at predetermined time intervals (so as to simulate tidal cycles) to microcosms partially immersed in temperature-controlled water baths illuminated by programmable luminaires (for diel light cycle simulation). Modifications to its original version were performed in order to: i) increase the number of pH and temperature (T °C) levels generated in the system that, in a factorial experimental design, broaden the range of OA-W scenarios simulated from four (two pH * two T °C levels) to twelve (three pH * four T °C levels); and ii) adapt the microcosms/tanks to the exposure of planktonic larvae (from 3 L-rectangular aquaria with automatic drainage by outflow pumps ﻿operated with digital timers, to inverted carboys with aeration and gravity drainage by outlet pipes with 100 µm-mesh filter-protected output, at such a height to allow a maximum interior volume of 3 L).

Synthetic saltwater was distributed by the four reservoirs of the ELSS (PRODAC Ocean Fish sea salt mixture dissolved, at 30 psu – dominant salinity of the habitat where netted whelk egg capsules were collected), in reverse osmosis −V2 Pure 75, TMC, UK– purified freshwater at least 24 h prior to use). Saltwater pH was manipulated in three of the four reservoirs to generate three distinct pH levels: control (pH_target_ 8.1) plus two acidified conditions of −0.3 and −0.5 pH units, corresponding to the interval of sea surface pH projected for the end of the 21^st^ century by the IPCC in the northern hemisphere under RCP8.5 scenario^11^, and in line with the available guidelines applied to OA research^45^. The fourth reservoir was used to keep unmanipulated saltwater to refill the remaining three as needed. Saltwater in all the reservoirs were recirculated for 15 min every two hours to promote water mobilization and to force pH adjustment.

Regarding T °C, the two water baths initially installed (see Fig. 1 in Coelho *et al*.^44^) were replaced by four of half of their size. Each new bath was equipped with a temperature control system similar to the original but scaled to its dimension. Four T °C levels were then generated: two control conditions –16 and 18 °C– corresponding, respectively, to the mean SST at the Ria de Aveiro estuarine system (NW Portugal) and the mean registered during *T. reticulata* spawning season at the site in the Ria de Aveiro where the specimens for this work were obtained^3^; plus two warming conditions −20 and 22 °C– that, according to the IPCC projection under RCP8.5 of a global increase in the SST of up to 4 °C^9,10^ correspond to end of century scenarios for controls 16 and 18 °C, respectively.

Each of the 12 OA-W scenarios generated was tested in duplicate. The Ria de Aveiro semi-diurnal tidal regime and the water renewal percentage at its central area^44,46^ were simulated by ~50% exposure medium exchange twice a day (manually at 9 am and 6 pm), by addition at the bottom of each tank through an inlet pipe, forcing the excess of medium to drain by gravity from the outlet pipe installed at the top. A 12h^L^:12h^D^ photoperiod was applied, at half of the light intensities considered by Coelho *et al*.^44^, as well as gentle aeration at 1 bubble seg^−1^, at intervals of 15 min during the day and 15 min every 2 h at night (reduction to compensate the logistical constraint of effecting media exchanges at 9 am and 6 pm, which implied a further 6 h stay of the media during the night period until the morning exchange).

**Figure 1:**
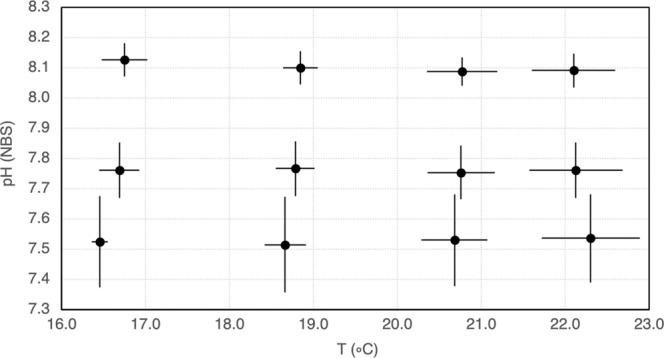
Twelve OA-W experimental scenarios generated during the 14 days trial corresponding to the temperature (T °C) and pH daily variation per treatment. Mean values and respective standard deviation of probe measurements of T °C (measured before media exchange) and pH (measured before and after media exchange) twice a day for 3 consecutive days from day 0 (T0) and once a week thereafter.

A pre-assay to monitor the diel cycles of pH and T °C generated in the system under such conditions was carried out and the respective control systems’ settings adjusted to avoid treatments’ overlapping and allow the predetermined scenarios to be obtained: pH controllers were set to 7.95, 7.35 and 6.95, and cooler/heater thermostats to 16/16, 17/18, 19/20 and 21/22 °C, generating the 12 different treatments plotted in Fig. 1.

### Sampling and endpoints

Egg capsules of *T. reticulata* were collected, by hand at the intertidal during low tide, inside the Ria de Aveiro (NW Portugal) at 40°38′34.65″N − 8°44′06.80″W. Once in the laboratory, egg capsules were kept acclimating for 3 days in 1 µm-filtered synthetic unmanipulated saltwater at 30 psu and 18 ± 1 °C. Following the protocol of Génio and co-workers^47^, mature egg capsules –containing larvae with well-developed eyes, statocysts and beating velum– were selected and ripped to release nearly-hatching veligers that were kept, for additional 24 h-acclimation, at a density of 2 larvae mL^−1^ and fed an appropriate microalgae blend: *Rhodomonas salina* (5000 cells mL^−1^), *Isochrysis galbana* (20000 cells mL^−1^) *Skeletonema costatum* (10000 cells mL^−1^) and *Tetraselmis chui* (500 cells mL^−1^), (diet based on the applied by Chatzinikolaou & Richardson^48^).

Right before the start of the exposure period (at day 0, T0), swimming veligers were collected in a 100 µm-sieve, back-washed into a 5 L-beaker to be counted, and aliquoted adequately to be randomly placed in the 24 tanks at a density of 1.6 larvae mL^−1^. Larvae were then exposed for 14 days to the 12 OA-W experimental scenarios, fed the microalgae blend described above, daily, after the late afternoon medium exchange in order to avoid pH imbalance due to photosynthesis during the day, and as larvae of marine bottom invertebrates are considerably more active in the water column during dark hours favoring feeding^49^.

Physico-chemical parameters of the exposure media (pH, T °C, salinity, total dissolved oxygen and carbonate chemistry) and biological endpoints (mortality, developmental stage, vertical migration, gut content, shell growth and integrity) were periodically assessed as follows.

#### Exposure media physico-chemical analysis

Probe measurements (Multi 3430 IDS, WTW, Germany) of pH (NBS scale) and T °C were recorded before and after both daily medium exchanges for three consecutive days from T0, to characterize the daily variations to which specimens would be exposed. Then, those parameters were periodically monitored, at least before one of the two daily medium exchanges, until day 14 (T14). Probe measurements of salinity (SAL) and total dissolved oxygen (DO) were recorded periodically, always before the morning medium exchange. Total alkalinity (TA) was determined by manual volumetric titration following Frommlet *et al*.^50^ protocol in 20 mL samples of 0.2 µm-filtered exposure medium collected at T0, and before and after the morning medium exchange at day 2 (T2) and T14 in one of the replicates per treatment. The values of TA and of pH, T °C and SAL read at the time of media sampling, were the inputs to calculate the CO_2_ partial pressure (*p*CO_2_), carbonate (CO_3_^2−^) ion concentration and the saturation states of calcite (Ω_Ca_) and aragonite (Ω_Ar_), using the Microsoft Excel macro CO2Sys v2.1^51^, with K1 and K2 carbonate dissociation constants from Mehrbach *et al*.^52^ refitted by Dickson & Millero^53^, and KSO_4_ from Dickson^54^.

#### Analysis of biological endpoints

Three sampling moments were carried out: at T2, to assess the randomness of the initial distribution of the veligers and mortality across treatments; at days 8 (T8) and 14 (T14), to assess all biological endpoints under study (mortality, developmental stage, vertical migration, gut content, shell growth and integrity).

At T2 veligers were collected in a 100 µm-sieve, back-washed into a beaker, suspended in 3 L of the respective exposure medium. The living larvae were counted in three aliquots of 5 mL per replicate (i.e., 6 per treatment) to determine survival and calculate mortality.

At T8 and T14, larvae were also collected in a 100 µm-sieve but suspended in a volume adjusted to the number of live individuals in each replicate. The migration test described by Braubach and co-workers^55^ to detect effects of neuroactive chemicals on the swimming ability of *Ilyanassa obsoleta* veligers was here applied with some adjustments. Two aliquots of 10 mL of larvae suspension per replicate (i.e., 4 per treatment) were aspirated into glass graduated pipettes and left standing vertically for 10 min, away from any direct light source (preliminary experiments indicated positive phototaxis and that a 10 min time period render reproducible results on the regular vertical distribution of *T. reticulata* veligers). Then, three fractions of each column were collected in a tripartite Petri dish –the bottom 2.5 mL, the middle 5 mL and the top 2.5 mL– and live veligers were counted per fraction to assess their vertical distribution, determine survival and calculate mortality. Of those larvae, 12 per replicate (i.e. 24 per treatment) were randomly selected and transferred to a 24-well plate (1 per well), to proceed with the analysis of further endpoints. Under a stereomicroscope (Leica S8 APO, Leica Microsystems, Switzerland), each specimen developmental stage was categorized into three (I to III) veliger stages following Zupo & Patti^56^ classification, and the presence or absence of shell and digesting food in each larvae gut were also registered. Then, and in addition to a representative sample of the initial (T0) pool of recently hatched larvae, 20 veligers per replicate were preserved in absolute ethanol to measure the shell length (SL), calculate growth rates, and analyse shell microstructural integrity under Scanning Electron Microscopy (SEM). Individuals were photographed under a stereomicroscope (Leica S8 APO equipped with a MC170 HD camera; Leica Microsystems, Switzerland) and the SL measured using Leica LAS measurement module. The same specimens were then prepared for SEM, following the protocol developed by Bednaršek *et al*.^57^ to characterize juvenile pteropod shell dissolution under acidity but adding an extra step of ethanol 70° overnight prior to the cleaning and drying procedure in order to rehydrate the samples, easing periostracum removal. Larval shells were observed and photographed using a benchtop Hitachi TM3030 (Hitachi High-Technologies Corporation, Japan) at 15 kV in MIX mode (simultaneous backscattered and secondary electrons imaging) with charge-up reduction. As unshelled larvae were registered at pH_target_ 7.5 treatments, other 12 veligers per replicate were relaxed in a solution 1:1 of the respective exposure medium and MgCl_2_ 7% in distilled water for 15 min, fixed in 2.5% glutaraldehyde in Millonig’s phosphate buffer and 2% osmium tetroxide in 1.25% sodium bicarbonate buffer, following Ellis *et al*.^58^ protocol up to dehydration (i.e., skipping decalcification, unnecessary in this case). Samples were then subject to critical point drying in order to image soft tissues in unshelled larvae through SEM. The SEM/EDS exam was performed using a High resolution (Schottky) Environmental Scanning Electron Microscope with X-Ray Microanalysis and Electron Backscattered Diffraction analysis: Quanta 400 FEG ESEM/EDAX Genesis X4M. Samples were coated with a Au/Pd thin film, by sputtering, using the SPI Module Sputter Coater equipment.

### Statistics

Permutational multivariate analysis of variance (PERMANOVA + add-on in PRIMER-E v7; Anderson *et al*.^59^) was used to test for significant effects of pH, T °C and the possible interaction between these factors for each endpoint analysed. For mortality, one value per replicate (typically the mean of three observations) was used in order to preserve a balanced PERMANOVA design. Vertical migration was tested using the mean of organisms in upper, mid and lower position on the water column. All other variables were tested per individual (12 larvae per replicate). Euclidean distance was applied to generate the resemblance matrix. A two-way crossed design (pH * T °C) was applied with a type III partitioning of the sums of squares and the permutation of residuals under a reduced model based on 9999 permutations to obtain the P-value. Prior to a significant PERMANOVA result, a pair-wise t test was used to understand which levels of the factor were responsible for the significant results.

## Results

### Experimental conditions

Figure 1 shows the 12 OA-W scenarios generated in the ELSS (mean temperature vs mean pH probe measurements ± standard deviation) and data on exposure media carbonate chemistry per treatment are compiled in Table 1. All treatments were found to be significantly different (Pseudo-F = 2965.8, p = 0.0001) and no significant difference between replicates from the same treatment was observed (p > 0.05). Salinity (30.14 ± 0.18) and dissolved oxygen (7.09 ± 0.309 mg L^−1^) were registered throughout the experiment, before the morning media exchange. Regarding these parameters, the longest media stay during the night did not imply critical conditions for larval survival. On the other hand, measurements performed before and after the daily medium exchanges characterize the variation of the system parameters to which the organisms were exposed during 24 h. Temperature varied ±1 °C with the exception of the initial 15 min equilibrium period, after media exchange, that showed a higher variation in upper temperatures (Fig. 1). The pH tended to increase over time until its re-establishment to target levels upon media exchange, having higher amplitudes at lower pHs (Fig. 1, Table 1). Saturation states of calcite (Ω_Ca_) and aragonite (Ω_Ar_) followed similar patterns to pH variation. Undersaturation was only registered at the most acidic treatments (pH_target_ 7.5): of aragonite (Ω_Ar_ < 1) was registered both “Before” and “After” media exchange while of calcite (Ω_Ca_ < 1) was only recorded “After” media exchange.

**Table 1:** Mean carbonate system parameters calculated from samples taken at T0, and “Before” and “After” the morning exchange of 50% of the exposure medium in one replicate per treatment at T2 and T14.

Treatment	Probe measurements					Titration		CO_2_Sys Excel macro							
T_target_	pH_target_	SAL	T	pH		TA		*p*CO_2_		CO_3_^2−^		ΩCa		ΩAr	
(°C)	(NBS)	(psu)	(°C)	(NBS)		(μmol kg-SW^−1^)		(μatm)		(μmol kg-SW^−1^)					
				Before	After	Before	After	Before	After	Before	After	Before	After	Before	After
16	8.1	30.1	16.7	8.18	8.17	2493	2566	447	477	167	164	4.11	4.04	2.62	2.57
	7.8	30.1	16.7	7.80	7.67	2615	2651	1240	1687	81	61	1.99	1.51	1.27	0.96
	7.5	30.0	16.5	7.57	7.36	2889	2644	2365	3548	56	31	1.38	**0.76**	**0.88**	**0.48**
18	8.1	30.1	18.9	8.11	8.12	2429	2582	527	539	151	162	3.74	3.99	2.39	2.55
	7.8	30.1	18.8	7.80	7.65	2566	2628	1245	1776	84	62	2.09	1.54	1.34	0.98
	7.5	30.0	18.5	7.58	7.35	2625	2661	2202	3745	54	32	1.34	**0.80**	**0.86**	**0.51**
20	8.1	30.1	21.0	8.11	8.11	2434	2615	543	576	159	169	3.95	4.17	2.54	2.68
	7.8	30.1	20.9	7.80	7.64	2615	2635	1285	1863	92	65	2.29	1.61	1.48	1.03
	7.5	30.0	20.7	7.59	7.35	2561	2498	2137	3622	57	32	1.43	**0.79**	**0.92**	**0.50**
22	8.1	30.1	21.7	8.14	8.09	2395	2553	486	596	171	168	4.25	4.18	2.74	2.70
	7.8	30.1	21.5	7.83	7.65	2620	2645	1196	1921	100	69	2.48	1.72	1.60	1.11
	7.5	29.9	21.7	7.67	7.35	2498	2547	1700	3828	68	34	1.68	**0.85**	1.09	**0.55**

The treatment column refers to target temperature (T_target_) and pH (pH_target_) levels selected as explained in the Materials and Methods section. Partial pressure of CO_2_ (*p*CO_2_), carbonate ion concentration (CO_3_^2−^) and saturation states of calcite (Ω_Ca_) and aragonite (Ω_Ar_) were derived from probe measurements of salinity (SAL), temperature (T) and pH, and from total alkalinity (TA) determined by volumetric titration^50^. Values for all carbonate species “After” media exchange were estimated by CO_2_Sys Excel macro^51^ from T_target_ levels as output parameter (since T_target_ was achieved after ca. 15 min from the 50% media exchange). Values in bold point out experimental conditions at which calcite and/or aragonite undersaturation were registered.

### Mortality

**Figure 2:**
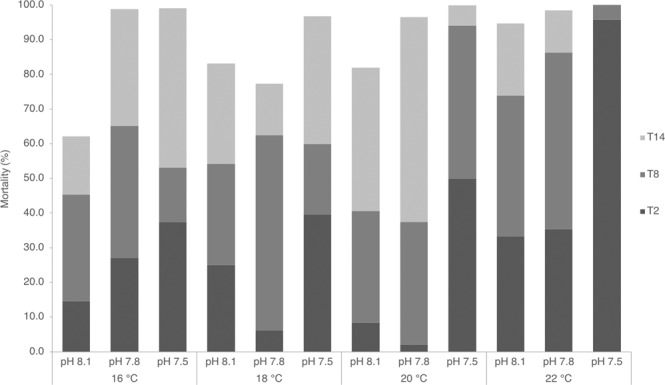
Cumulative mortality calculated from the mean survival per treatment throughout the experiment at 2 (T2), 8 (T8) and 14 (T14) days of exposure to the 12 OA-W scenarios: 3 pH levels (8.1, 7.8 and 7.5) at 4 different temperatures (16, 18, 20 and 22 °C).

Figure 2 represents the cumulative mortality of *T. reticulata* veligers, per experimental treatment, throughout the 14 days of exposure. At T2, mortality appeared to be driven by the pH only (Pseudo-F = 8.0715, p = 0.0073) with higher mortalities observed at the most acidic treatments (pH~7.5) and significantly differing from the controls (pH~8.1; t test=3,8953, p = 0.0063) and the intermediate pH scenarios (pH~7.8; t test=3,852, p = 0.0062). At T8, not only the pH lessening (Pseudo-F = 4.2679, p = 0.038) but mostly the increasing temperature (Pseudo-F = 7.1707, p = 0.0059) were found to account for higher larval mortality. The highest tested temperature, 22 °C, was significantly different from all others (22 vs 20 °C: t test=3.7021, p = 0.0145; 22 vs 18 °C: t test=3.6398, p = 0.011; 22 vs 16 °C: t test=5.1176, p = 0.0033) contributing with the highest mortality while the difference between pH levels was only significant between 7.5 and 8.1 treatments (t test =2.6867, p = 0.0296) across temperatures. At T14 temperature and pH interactively affected survival (Pseudo-F = 11.723, p = 0.0002). In this case, pairwise tests within the three pH levels and the four temperatures were performed to understand the significant effects of their interaction. Significant lower mortalities were observed within the control pH at 16 °C whilst no significant effects were observed in between temperatures from the two acidified conditions. Comparisons in between temperatures showed significant differences between pH controls (8.1) and the two other treatments but only for both temperature 16 and 20 °C meaning that, at these two temperatures, significant lower mortalities were observed at the control pH treatments.

### Larval development

**Figure 3:**
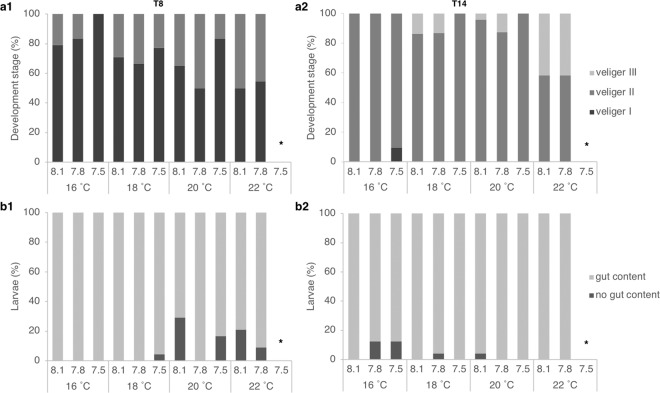
*T. reticulata* larval culture endpoints (shown in different rows: (**a**) percentage of veligers at development stage I, II or III; (**b**) percentage of veligers with or without visible food content inside the gut) by time point (shown in different columns: 1. at 8 days of exposure, T8; 2. at 14 days of exposure, T14). *Treatment without survivors (100% mortality).

The performance of *T. reticulata* veligers was assessed through the evaluation of their development stage (intimately related with their position in the water column, i.e., with their vertical migration) and their feeding state at both T8 and T14 (Fig. 3). Replicates within the same treatment never diverged significantly (p = 0.4485 and p = 0.2068 for T8 and T14, respectively). At T8, larval development (Fig. 3a1) was significantly affected both by increasing temperature (Pseudo-F = 4.8099, p = 0.0033) and decreasing pH (Pseudo-F = 3.9199, p = 0.022). The lowest temperature, 16 °C, differed from all others (16 vs 18 °C: t test=2.3857, p = 0.0164; 16 vs 20 °C: t test=3.1916, p = 0.0017; 16 vs 22 °C: t test=3.4464, p = 0.001) with the majority of larvae on the first stage of development (less than 20% of stage II veligers on the pH 8.1 and the only temperature without stage II veligers in the most acid treatment, 7.5). All other temperatures did not display a substantial difference among them. The significant effect of pH was achieved through the difference between the most acidic treatment 7.5 and the other two (7.5 vs 7.8: t test=2.883, p = 0.0048; 7.5 vs 8.1: t test=2.1586, p = 0.035). At T14 (Fig. 3a2), temperature was found to be the main driver for development (Pseudo-F = 12.495, p = 0.0001), with larvae at 22 °C significantly more advanced than those at lower temperatures e.g. 40% of larvae at stage III at 22 °C against ~10% at 18 and 20 °C and none at 16 °C. Besides, significant differences were also found between larvae raised at 16 and 18 °C but not between 16 and 20 or 18 and 20 °C.

In this study, vertical migration of *T. reticulata* was not significantly affected by the pH, temperature or their interaction at T8 or at T14. However, pH had a significant effect on veligers’ gut content (Pseudo-F = 3.916, p = 0.0162) and, even greater, of the temperature (Pseudo-F = 7.2252, p = 0.0004) at T8 (Fig. 3b1). Approximately 20% of the larvae from treatments 20 and 22 °C did not present digesting food inside the gut. Presence of gut content did not significantly differ between larvae reared at 20 and 22 °C or between 16 and 18 °C. However, significant differences were observed between 16 vs 20 °C (t test=3.578, p = 0.0004), 16 vs 22 °C (t test=3.2057, p = 0.0007), 18 vs 20 °C (t test=2.9732, p = 0.0039) and 18 vs 22 °C (t test=2.9433, p = 0.003). Despite the pH effect, treatments 8.1 and 7.5 were not considerably different (p > 0.05). Nonetheless, the differences between all other treatments were statistically significant (7.5 vs 7.8: t test=2.1351, p = 0.0322 and 7.8 vs 8.1: t test=2.7357, p = 0.0061). At T14 no significant effects neither of pH nor temperature (or their interaction) were observed (Fig. 3b2).

### Larval growth, shell integrity and loss

**Figure 4:**
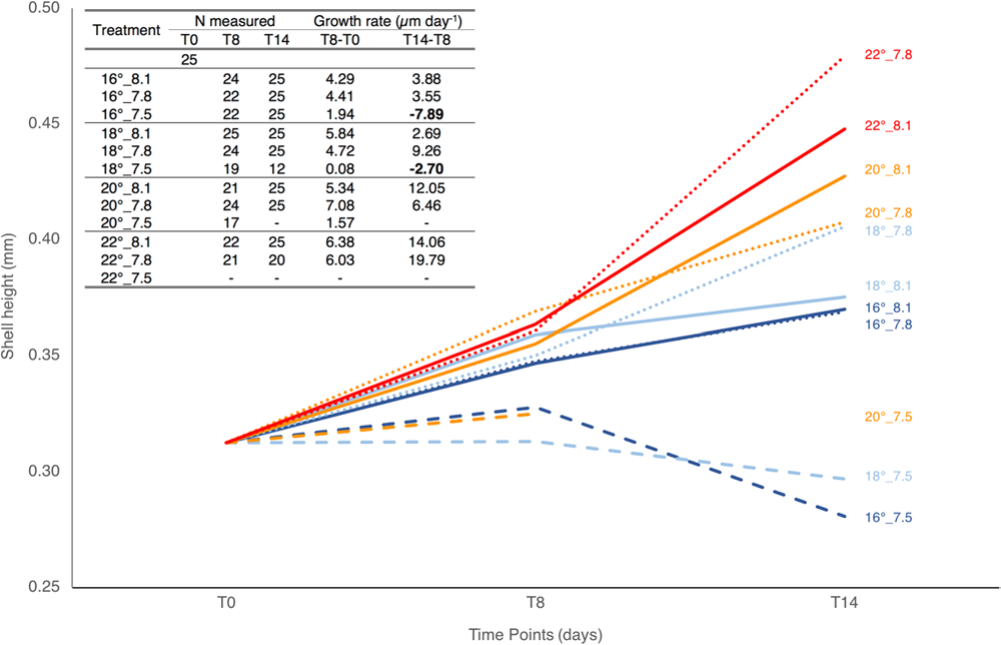
*T. reticulata* mean shell length (SL) evolution across time points, namely at the beginning of the experiment (T0), after 8 (T8) and 14 (T14) days of exposure. Solid, dotted and dashed lines correspond to the pH_target_ 8.1 (control), 7.8 and 7.5, respectively. Different colours correspond to different temperatures (16 °C: dark blue; 18 °C: light blue; 20 °C: orange; 22 °C: red). Lines are labelled with the reference to the respective treatment (in the format temperature_pH) on the right side of the graph. The insert (table) on the upper left corner compiles the number of animals measured (N measured) per treatment and time point, and the growth rate (expressed in µm day^−1^) on the first (T8-T0) and second (T14-T8) week of exposure. Bold values evidence negative growth rate (reflecting shell dissolution).

**Figure 5:**
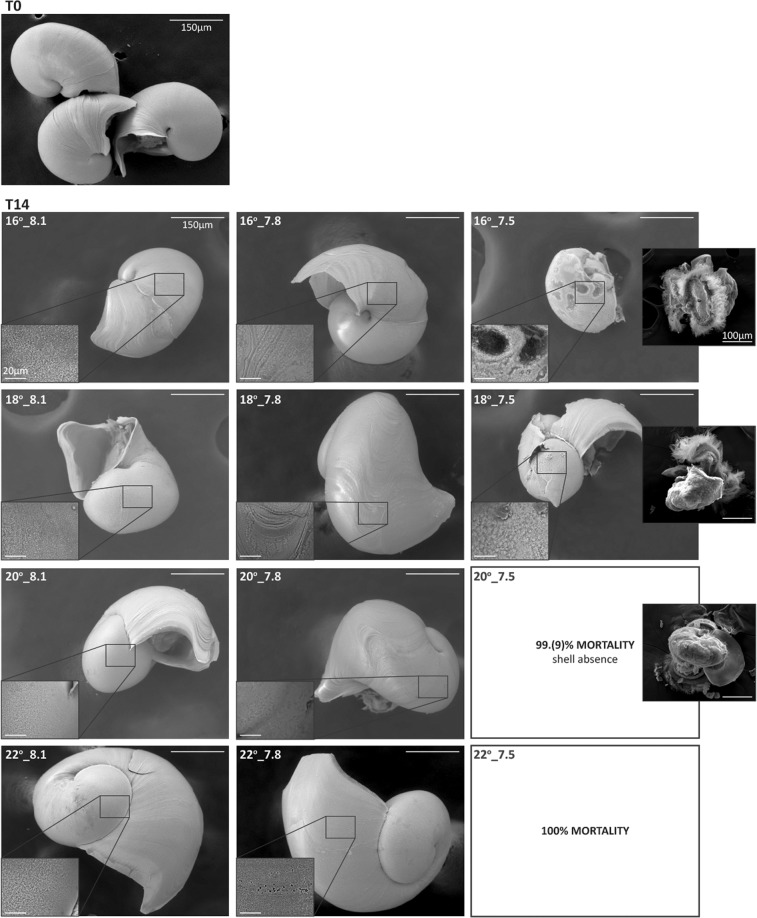
*T. reticulata* under Scanning Electron Microscopy (SEM). Aspect of the shell of veligers collected at T0 (top image) and at T14 after exposure to 12 OA-W experimental scenarios (indicated at the top left of each image; scale bar = 150 μm). Inserts in the lower left corner of each image are shells’ surface (scale bar = 20 μm). Pictures of unshelled veligers after critical point drying, detected only at pH_target_ 7.5 scenarios, are shown overlaid on the right side of the image of the respective treatment (scale bar = 100 μm).

*T. reticulata* shell length (SL) was measured at T0, T8 and T14 in order to calculate growth rates per treatment (Fig. 4) and define if and how pH and temperature affect this species’ growth in early life. After 8 days of exposure a significant effect of the pH was registered (Pseudo-F = 17.763, p = 0.0001). Veligers at the most acidic treatments had significant lower SL than those at the other pH levels (7.5 vs 8.1: t test = 4.5655, p = 0.0002 and 7.5 vs 7.8: t test = 4.8417, p = 0.0001). This is reflected on the lower growth rates recorded at the most acidic experimental scenarios (Fig. 4). Despite lower, growth rates at the most acidic treatments were positive at T8, so no evidence of shell dissolution was perceived after a week of exposure. However, negative growth rates were registered at T14 at the most acidic treatments (see bold values in the insert in Fig. 4). Moreover, a significant interaction of temperature and pH was observed at this time point (Pseudo-F = 2.9759, p = 0.0201), being evident, in Fig. 4, that organisms increased in size with the temperature rise throughout the 14 days exposure, but only under less acidic conditions (i.e., for pH ≥7.8). In fact, no significant differences in SL were found between treatments at pH 8.1 and 7.8 at both 20 and 22 °C. However, at 16 °C, the most acidic condition (7.5) rendered expressively smaller animals, being significantly different from the other two pH treatments (with no differences between the control pH and the 7.8). At 18 °C, all treatments were significantly different from each other, and the specimens under pH~7.8 exhibited the highest mean shell size. Comparisons within the same pH level revealed that, under control 8.1, only the two most extreme treatments were not significantly different (i.e., 16 vs 18 °C and 20 vs 22 °C). Within pH~7.8, SL means differed significantly between treatments with the single exception of 18 vs 20 °C, and no significances were achieved within the two existing treatments at pH~7.5 at the end of the experiment. Negative growth rates registered at T14 at pH_target_ 7.5 treatments called the attention for shell dissolution. In fact, results from SEM on veligers collected at the end of the experiment (Fig. 5) revealed slightly increased porosity and fragility of the growing edges in shells built under decreased CaCO_3_ supersaturation (i.e., under pH~7.8): the type I dissolution of Bednaršek *et al*.^57^. However, severe dissolution, of types II and III, was evident in shells built at the most acidic, undersaturated treatments. Under these conditions, extensive damage was observed: surfaces covered by numerous dissolved patches, compromising shell integrity and making it of an extreme frailness. In fact, not only shell dissolution but also the occurrence of anatomically normal veligers lacking shells was observed under the most acidic treatments during the experiment.

**Figure 6:**
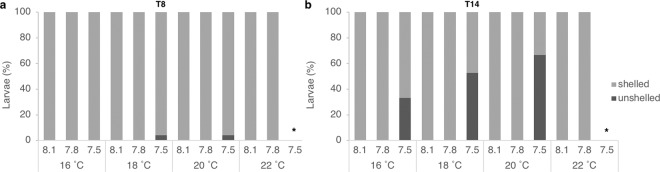
*T. reticulata* shell loss. Percentage of veligers with or without shell (shelled or unshelled, respectively) registered (**a**) at 8 (T8) and (**b**) at 14 (T14) days of exposure. *Treatment without survivors (100% mortality).

The number of unshelled veligers (i.e., specimens without shell but actively swimming) registered throughout exposure is plotted in Fig. 6. This occurrence was first recorded at T8, only at 18 and 20 °C and in relatively low percentages (4% of the specimens analysed in each of those treatments). Nevertheless, the effect of pH on shell loss was already significant at this time point (Pseudo-F = 2.4839, p = 0.0343). At T14, increasing percentages of unshelled veligers were registered, only at the most acidic condition, varying from 33 to 67% with warming (from 16–20 °C). Despite apparent, this increasing trend with warming was not significant and only the strong significant effect of the pH on the shell loss condition was proved (Pseudo-F = 48.079, p = 0.0001; 7.5 vs 8.1: t test = 7.3403, p = 0.0001 and 7.5 vs 7.8: t test = 6.9637, p = 0.0001).

## Discussion

### Effects of OA-W on *T. reticulata* larval development and survival

Certain molluscs’ species have complex life cycles in which different stages of development need to adapt to survive in distinct ecological niches. This must be complemented by anatomical, physiological and behavioural adaptations that differ across development stages^55^. Previous studies have shown that certain biological responses are likely to differ across such stages^43,60,61^. *T. reticulata* has a planktonic larval phase that develop into four stages (defined by Zupo & Patti^56^), reaching competence to metamorphose and settle, undergoing irreversible alterations in order to acquire a benthic life style. This work shows that each endpoint was differently affected by pH and temperature at a specific time of exposure during the early larval phase. The analysis of the larvae development revealed a significant effect of temperature and pH (but not of their interaction) after 8 days of exposure, whilst after 14 days only temperature was found to exert a significant effect. It is widely recognized that temperature has a major influence on the development and growth of marine invertebrate larvae. As reported previously for other molluscs, organisms seem to “evolve faster” under higher temperatures reaching upper development stages much quicker than those at lowest temperatures^39^. However, before and beyond the thermal tolerance range of a certain species, a metabolic depression is likely to occur^34,60,62^. In the present study, *T. reticulata* larvae also experienced a faster development with warming but T8 veligers disclosed a delayed development in the most acidic pH when compared to controls at the same temperatures. This tendency was also apparent at T14, although the results were not statistically conclusive probably due to extreme mortalities observed in the most acidic treatments (~100% in both replicates of treatment 7.5_20 °C and 100% in 7.5_22 °C). Delay in larval development due to ocean acidification was already reported on other marine invertebrate early life stages^23,60,63^, including gastropods^31^. This delay has been suggested to be a primary consequence of altered energy budget under abiotic stress regimes^64^. Species with an open circulatory system, such as gastropods, have a low capacity to maintain the acid-base balance of their extracellular fluid under acidified conditions, owing to their low metabolic rates and large volumes of extracellular fluid^34^. As a substantial part of the metabolic energy may be required for acid-base regulation, they are vulnerable to developmental delays, hindered growth, hypercapnia and even death, due to energy trade-offs^34,37^.

Survival was also affected by pH and temperature, individually or in combination, across time points. After only two days of exposure, lowering pH caused significant increased mortalities across temperatures, which reach the most notorious expression in pH 7.5. A similar trend was observed after 8 and 14 days of exposure. Such results might have occurred due to the above-mentioned energy trade-offs or even hypercapnia, at low pH, and are consistent with previous reports of reduced survival of early calcifying larvae when they are exposed to enriched aqueous CO_2_ concentrations^26,31,65^. The highest temperature (22 °C) caused an additional stress leading to an enhanced mortality. By coincidence, at T8, larvae exposed to 20 and 22 °C were malnourished (see results on veligers’ gut content on *Larval development* section). The concentration of algae was monitored in the experiments to assure food was kept at *ad libitum* levels, so the stress induced by higher temperatures must have disturbed the feeding performance of the larvae. Likewise, the existence of malnourished larvae at the most acidic pH (7.5) suggests that pH may affect feeding, as previously reported in the gastropod *Concholepas concholepas*^66^. An inability to feed effectively will negatively impact metabolic functions that may ultimately result in decreased survival^67^. Malnourishment was only observed at T8, and survivors were evaluated as well-nourished on the following time point (T14).

### Effects of OA-W on shell growth and integrity

Marine calcifiers shell is a primary defense against adverse environmental conditions. In gastropod larvae, shell formation begins at late trochophore stage and is termed protoconch I, followed by the secretion of protoconch II during the veliger stage (see review by Marin *et al*.^68^). In the present study, the exposure to the different treatments started at 48 h post hatching, moment when veligers should be most likely secreting protoconch II. It is accepted that amorphous calcium carbonate (CaCO_3_) is the first polymorph of the mineral deposited by mollusc veligers, and is the main constituent of their shells^69^. Amorphous CaCO_3_ is the precursor of crystalline polymorphs, which, in gastropods, is known to be mainly aragonite^69,70^. Because amorphous CaCO_3_ and aragonite are relatively soluble, when compared to calcite, shells from early larval stages are vulnerable to acidification, especially when carbonate saturation (Ω) is reduced (≤1). In fact, after 8 days of exposure veligers under the most acidic pH treatments (7.5) –the only undergoing Ω_Ar_ < 1 (Table 1)– showed reduced shell length (SL) and, thus, decreased growth rates (Fig. 4). Aragonite undersaturation most probably increased the energy cost of calcification^71,72^ hindering shell growth and possibly altering the chemical properties of veligers’ shells^17,73,74^. Such result is not a surprise as reduction in shell size and/or growth due to ocean acidification has been frequently reported (see reviews by Bednaršek *et al*.^75^ and Kawahata *et al*.^76^). Even so, as temperature is known to accelerate development and increase growth, our expectation was that the effect of acidification on growth could be balanced, compensated by warming, which did not occur at an early stage (i.e. at 8 days of exposure).

After 14 days of exposure an interaction between pH and temperature was evident. In general, higher temperatures prompted greater growth while the decreasing pH hampered it. Surviving veligers at lowest pH showed negative growth rates, with the lowest temperature showing the most negative value. Low pH led to shell dissolution, as revealed by the microstructural study of larval shells under SEM (see Fig. 5). There was a severe shell damage and frailness under types II and III dissolution, which explain the estimated negative growth rates (Fig. 4). Ries and co-workers^21^ have shown a strong effect of OA-W in the dissolution of biogenic carbonates, especially in shells of more soluble polymorphs of CaCO_3_ built under undersaturation (in respect to both aragonite and calcite). All our other treatments showed positive growth, with greater shell sizes registered at higher temperatures. Curiously, and despite the type I dissolution detected at larvae building shells under pH 7.8 (a condition close but still above CaCO_3_ undersaturation), these larvae grew significantly faster (9.26 µm day^−1^) than those in control (2.69 µm day^−1^) at 18 °C and pH 8.1. Further research must be undertaken to explain this phenomenon, which is not the first time to be reported. Wood and co-workers^77^ have demonstrated that some organisms, as the ophiuroid brittlestar *Amphiura filiformis*, can increase both their metabolism and calcification rate in order to compensate seawater acidity. However, the cost of this upregulation was a trade-off between skeletal integrity (increased calcification) against reduced muscle mass.

Beyond the evidences of shell dissolution under acidity presented here (Fig. 5), shell loss was the most striking effect of exposure to the most acidic and undersaturated scenarios. This condition was observed exclusively under pH_target_ 7.5 treatments, with a significant effect of pH on shell loss at both time points (T8 and T14; Fig. 6). Nonetheless, despite the apparent intensification of the condition with warming at T14 (see Fig. 6), this could not be statistically confirmed, possibly due to the reduced number of animals available at 20 °C (only 3 survivors, 2 of which unshelled). The shell loss was not due to an abnormal secretion, or even non-secretion of the shell, as reported previously for abalone species^23,26,31^, nor to their complete dissolution as in pteropods^78^. Instead, we observed the literal loss “by dropping” the shell, as a whole, with veligers swimming while their shells were lying empty on the bottom of the tanks. After this striking observation, shell loss “by dropping” was confirmed in a sequent trial in which veligers were individualized in 24-wells plates and exposed to pH~7.5 (Oliveira *et al*.^79^). Byrne and co-workers^23^ have reported the occurrence of unshelled abalone specimens at pH 7.8, which was related with a sharp fall in the aragonite saturation state, potentially exacerbated by a metabolism suppression due to hypercapnia. Further reports of specimens lacking shells were performed by Crim *et al*.^26^ and Wessel *et al*.^31^ on organisms of the same *Haliotis* genera. Extreme shell dissolution has also been described to occur with pteropods^78,80^. Shell loss would lessen the chances of survival in nature since this structure plays essential roles in feeding, buoyancy control, pH regulation and defense against predation^78,80^.

The negative impacts of OA-W documented here, especially those occurring at the most extreme scenarios –lower pH and highest temperature– predicted by the IPCC, can have severe effects on *T. reticulata* populations. Even considering that adaptive processes of this species to OA-W cannot be disregarded, this study shows the extreme vulnerability of *T. reticulata* larvae to the concomitant effects of ocean acidification and warming. Our results highlight the risk of climate change to marine gastropod molluscs and call for urgent mitigation actions to reduce emissions of CO_2_ and other GHG.

## Data Availability

The dataset generated during the current study are available from the corresponding author on reasonable request.
